# Induced Pluripotent Stem Cell-Based Platforms for Cardiac-Related Pain Research: Molecular Mechanisms, Experimental Models, and Therapeutic Applications

**DOI:** 10.3390/ijms27167333

**Published:** 2026-08-17

**Authors:** Boya Liao, Aaron Kin-ho Lee, Zheng Gu, Fei Meng, Jiangwei Wu, Jinghan Yang, Stanley Sau-ching Wong

**Affiliations:** Department of Anaesthesiology, School of Clinical Medicine, Li Ka Shing Faculty of Medicine, The University of Hong Kong, Hong Kong, China; leeaaron@hku.hk (A.K.-h.L.); zhenggu996@gmail.com (Z.G.); fmeng@hku.hk (F.M.); jwwuhk@connect.hku.hk (J.W.); yangjinghan@connect.hku.hk (J.Y.)

**Keywords:** induced pluripotent stem cells, cardiac pain, angina, nociceptors, cardiomyocytes

## Abstract

Cardiac-related pain, including angina pectoris and pain accompanying myocardial infarction, remains clinically important, and existing treatments are inadequate for some patients. Animal and primary-tissue studies provide important mechanistic evidence, but species differences and limited access to patient-matched human neuro-cardiac tissue constrain translation. This review synthesizes the following separate advances relevant to future human iPSC-based cardiac-pain modeling: molecular mechanisms that may be reconstructed in vitro; platforms ranging from two-dimensional cultures to proposed sensory-innervated three-dimensional and microfluidic systems; and potential applications in target validation, patient-specific modeling, and analgesic screening. We distinguish direct human iPSC evidence from findings obtained in animal, primary-cell, cardiac-only, autonomic-neuron, or non-cardiac pain models. We also discuss maturation, standardization, and regulatory challenges. iPSC technologies may complement existing models and support mechanism-focused, patient-stratified research, but direct validation in human sensory–neuron–cardiac systems remains limited.

## 1. Introduction

Coronary heart disease and acute myocardial infarction impose a substantial population burden [1], while persistent angina can markedly impair the quality of life and increase healthcare use [2]. Despite contemporary anti-anginal therapy and revascularization, 2–24% of patients with coronary artery disease report daily or weekly angina; a smaller subset meets criteria for refractory angina [2]. Silent myocardial ischemia may delay clinical recognition and is associated with adverse outcomes [3]. Cardiac ischemia can generate or release candidate algogenic signals such as bradykinin and protons [4]. Purinergic mediators, including adenosine and ATP, participate in cardiac signaling [5]. ATP can activate cardiac sympathetic afferents during myocardial ischemia [6], whereas bradykinin can activate or sensitize peripheral nociceptors in the experimental contexts reviewed [7]. Oxidative-stress and neurotrophic mechanisms are considered separately below with explicit model and species qualifications. Peripheral-afferent excitability is shaped by voltage-gated sodium and transient-receptor-potential channels [8]; cardiac sensory neurons also exhibit acid-evoked currents mediated predominantly by acid-sensing ion channels [9]. This mechanistic heterogeneity, together with visceral-somatic convergence and supraspinal modulation, may contribute to interindividual variation in cardiac-pain presentation.

Progress toward mechanism-based cardiac analgesics is limited in part by differences between experimental animal models and human cardiac-injury and pain biology [10,11]. Comparative studies have documented species-dependent differences in sensory–neuron electrophysiology [12]. P2X3 desensitization is a major determinant of receptor signaling [13], and direct human–rat comparisons show differences in P2X3 kinetics and pharmacological properties [14]; these observations warrant caution when extrapolating preclinical findings. Standard two-dimensional cardiac cultures lack the multicellular three-dimensional architecture and perfusable vasculature of native tissue [15]. Conventional monocultures also cannot reproduce reciprocal cardiomyocyte–autonomic–neuron interactions examined in dedicated co-culture systems [16]. Primary human DRG neurons and myocardial tissue provide valuable adult-tissue benchmarks but are limited by donor scarcity, tissue quality, and difficulty generating scalable patient-matched preparations. iPSC-derived cells offer renewable and genetically tractable complementary models, albeit with important maturation limitations.

Human-induced pluripotent stem cell (iPSC) technologies provide complementary human-cell-based components for this research. Human iPSCs can be differentiated into cardiomyocytes and sympathetic neurons [16], as well as nociceptive sensory neurons [17,18,19]. Published neuro–cardiac combinations include two-dimensional co-cultures and compartmentalized devices [20,21,22,23]. Published cardiac organoids and microtissues predominantly contain cardiac and supporting lineages [24,25,26,27]; sympathetic-neuron-innervated heart assembloids have been reported in a preprint [28]. Sensory–neuron innervation of cardiac organoids remains prospective. Human iPSC-nociceptor studies have used patch-clamp electrophysiology [29], multi-electrode arrays [18], calcium imaging [30,31], and transcriptomic profiling [17]. Isogenic SCN9A perturbation in human iPSC-derived nociceptors illustrates how gene editing can test a candidate pain pathway in a defined cellular context [29]; broader opportunities for nociceptor-selective treatment development have been reviewed [32]. Patient-specific iPSC lines enable investigation of genetic determinants underlying interindividual differences in pain susceptibility [19] and provide a platform for evaluating differential analgesic responses [33]. Whether these models can reliably capture epigenetic determinants or guide precision analgesic treatment remains to be established.

**Figure 1:**
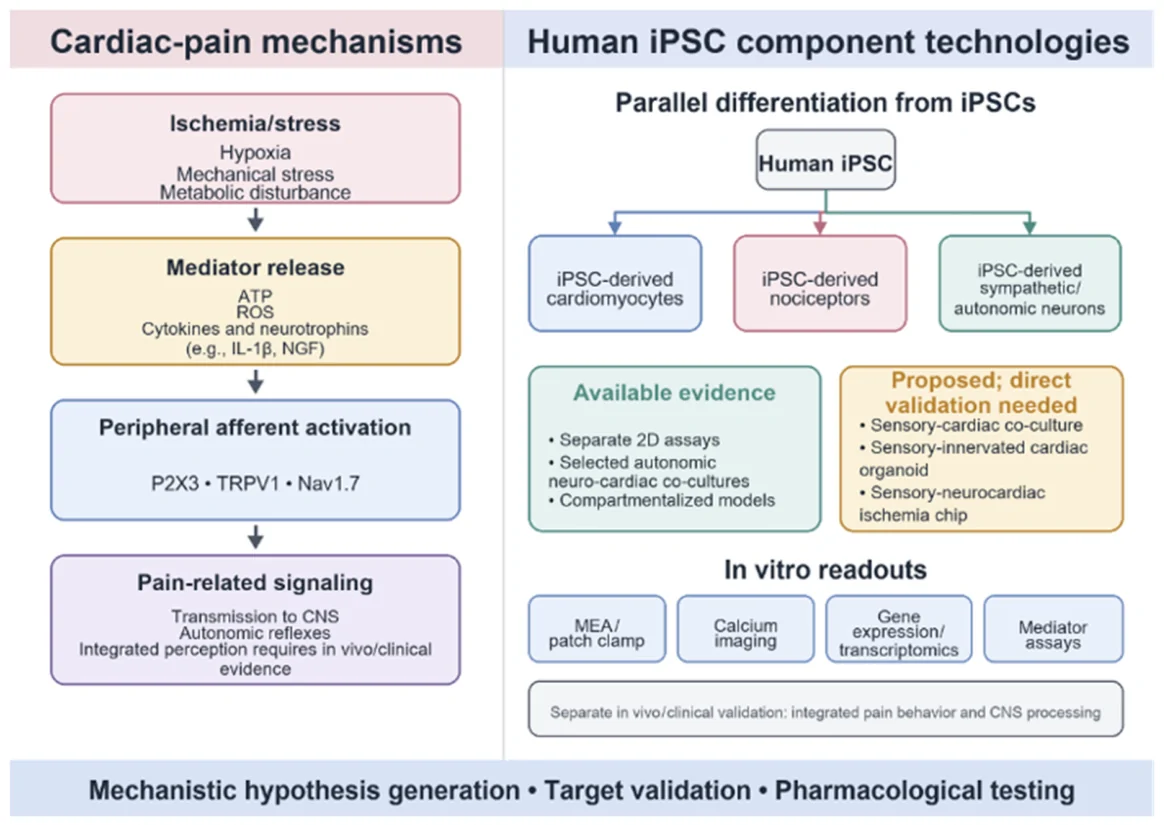
Component technologies and proposed human iPSC strategies for cardiac-related pain research.

This review evaluates separate advances relevant to future iPSC-based models of cardiac pain, with emphasis on species and model boundaries. We integrate key algogenic pathways with available two-dimensional, autonomic neuro-cardiac, cardiac-organoid, and microfluidic technologies, and identify proposed sensory–neuron–cardiac configurations that require direct validation (Figure 1). We then assess potential applications in target validation and analgesic screening while distinguishing established results from prospective patient-stratified uses.

Schematic overview of the cardiac-pain axis and experimental strategies relevant to future human iPSC modeling is provided. Myocardial ischemia and related stressors can generate candidate algogenic mediators that act on cardiac-afferent pathways. Human iPSC-derived cardiomyocytes and nociceptors can be studied separately, whereas published neuro-cardiac co-culture and compartmentalized models predominantly use sympathetic or autonomic neurons. Sensory-neuron-innervated cardiac organoids and sensory-neurocardiac ischemia-on-chip systems remain proposed extensions. Representative readouts include multielectrode-array recording, calcium imaging, patch-clamp electrophysiology, gene-expression analysis, and mediator measurements. The diagram distinguishes available component technologies from cardiac-pain models that still require direct validation.

## 2. Molecular Mechanisms of Cardiac-Related Pain and Their Interrogation in iPSC-Based Systems

Understanding cardiac pain requires consideration of algogenic signals generated during ischemia, nociceptor ion-channel plasticity, and neuro-cardiac signaling. This section examines pathways relevant to cardiac nociception using evidence from human, animal, and non-cardiac pain models and evaluates which components can be interrogated in human iPSC-derived cells. Direct evidence from validated human iPSC sensory–neuron–cardiac systems remains limited.

### 2.1. Purinergic Signaling: ATP Release and P2X3 Receptor Activation

Adenosine triphosphate (ATP) released during myocardial ischemia can activate P2X- and P2Y-receptor signaling in cardiac afferent pathways [5]. Ectonucleotidases subsequently degrade ATP to adenosine, and adenosine administration can evoke angina-like pain in humans [5]. A2-receptor signaling also influences vascular responses [34], while the net nociceptive effect depends on receptor subtype and experimental context [5]. The contribution of an ATP-to-adenosine sequence to sustained or refractory angina remains hypothetical. Direct human–rat comparisons show species-dependent differences in P2X3 receptor kinetics and pharmacological properties [14], supporting explicit species qualification when interpreting preclinical purinergic studies.

Subsets of mature iPSC-derived sensory neurons show calcium responses to ATP or the P2X3-preferring agonist α,β-methylene ATP [30,31]; separate MEA studies demonstrate pharmacologically evoked neuronal activity [18,35,36]. Direct equivalence to adult primary human DRG neurons should not be assumed. A multiwell MEA assay reported robust Z′-factors exceeding 0.5 under selected cell-density and pharmacological-control conditions [35]. In the cardiac context, this platform could be adapted to quantify ATP-evoked nociceptor firing and the effects of P2X3 blockade. Although gefapixant has demonstrated clinical activity in refractory or unexplained chronic cough, its efficacy in cardiac pain has not been established [37].

Co-culture of iPSC-derived nociceptors with iPSC-cardiomyocytes subjected to hypoxia-glucose deprivation is a proposed reductionist model of ischemia-associated cardiac afferent signaling. In such a system, extracellular ATP measurements together with apyrase or a P2X3 antagonist could test whether cardiomyocyte-derived ATP contributes causally to nociceptor activation. The biological rationale includes a rat study reporting myocardial-ischemia-related P2X3 signaling in stellate-ganglion neurons [38]; that study did not use human iPSC-derived cells.

### 2.2. Inflammatory Mediators and Oxidative Stress

Bradykinin and endothelin-1 can modulate afferent sensory-neuron calcium signaling through B2- and ETA-receptor pathways, although the downstream responses are not identical [39]. In rodent sensory-neuron studies, IL-1β increases excitability through p38-dependent modulation of tetrodotoxin-resistant sodium currents [40], NGF activates p38 MAPK and increases TRPV1 abundance [41], and TNF-α enhances P2X3 currents through p38 MAPK [42]. These findings support candidate sensitization pathways but do not establish their combined operation in human cardiac afferents. Spatial transcriptomic comparison of human and mouse DRG has identified species-divergent expression patterns across nociceptor subsets and pharmacological targets [43], supporting caution when extrapolating individual rodent inflammatory mechanisms.

In a mouse hindlimb ischaemia–reperfusion model, hypoxia sensitized TRPA1 to reactive oxygen species; whether this mechanism operates in human cardiac afferents remains unproven [44]. Human iPSC-derived nociceptors are established tools for mechanistic pain studies [45,46], but applying them to ischemia-associated cardiomyocyte-derived factors is a proposed cardiac-specific extension rather than a result demonstrated in those references. ATP can activate cardiac sympathetic afferents during myocardial ischemia [6]; bradykinin [7] and NGF [47] can activate or sensitize nociceptive pathways in the experimental contexts reported in those sources. Apyrase, neutralizing antibodies, and receptor antagonists are proposed interventions for testing the relative contributions of these mediators in a future human cardiac co-culture.

To further delineate the role of cardiomyocyte-derived inflammatory signaling in cardiac nociception, a human iPSC-based co-culture paradigm using conditioned medium from ischemia-like cardiomyocytes applied to nociceptor-like sensory neurons, combined with neutralizing antibody blockade of specific soluble mediators, could offer a tractable strategy for mechanistic validation.

### 2.3. Ion Channel Remodeling and Electrophysiological Plasticity

Voltage-gated sodium channels, particularly Nav1.7 (SCN9A), Nav1.8 (SCN10A), and Nav1.9 (SCN11A), are established regulators of nociceptor excitability and therefore candidate mechanisms for cardiac-pain research [48]. Nav1.7 contributes to action-potential threshold, and inflammatory injury can alter nociceptor channel expression and excitability [49]. Human loss-of-function SCN9A mutations can cause partial congenital insensitivity to pain [50] or complete congenital inability to experience pain [51], whereas gain-of-function variants are associated with inherited pain disorders such as erythromelalgia and paroxysmal extreme pain disorder [52]. These complementary human genetic findings establish Nav1.7 as a pain-biology target, but do not by themselves prove that pharmacological Nav1.7 inhibition will be clinically effective. Indeed, several Nav1.7-targeting compounds have shown limited clinical efficacy [53].

iPSC-derived nociceptors express SCN9A/Nav1.7 and SCN10A/Nav1.8 and display TTX-sensitive and TTX-resistant sodium currents, although their channel repertoire and gating retain developmental differences from adult primary human DRG neurons [54]. CRISPR-mediated SCN9A knockout increases rheobase and reduces repetitive firing in response to suprathreshold current injection [29]. These platforms can be used to investigate Nav1.7-dependent excitability, but they cannot by themselves determine why clinical inhibitors have shown inconsistent analgesic efficacy [53]. In one human iPSC-nociceptor study, 40–67% of cells responded to capsaicin, and functional responses involving selected ionotropic receptors and voltage-gated channels were documented [55].

### 2.4. Neuro-Cardiac Bidirectional Signaling

Myocardial injury can engage neuro-cardiac communication. In patients with unstable angina, an increased coronary sinus-aortic BDNF gradient is consistent with coronary vascular release [56]. In experimental myocardial infarction, a heart-to-brain afferent pathway increases central and circulating BDNF and limits adverse remodeling [57]. In a separate mouse nerve-injury model, BDNF-TrkB signaling reduced presynaptic GABAergic inhibition and contributed to neuropathic-pain induction [58]; this is non-cardiac evidence rather than direct proof in cardiac afferents. After experimental myocardial infarction in rats, increased NGF is associated with sympathetic hyperinnervation [59], while NGF can sensitize nociceptors in non-cardiac pain contexts [47]. Cardiac afferent activation also participates in autonomic reflexes, but their direction and clinical consequences depend on experimental and disease context [4]. Sensory neuron-derived CGRP has vasodilatory and potentially cardioprotective actions [60].

Direct validation of bidirectional signaling in human iPSC-derived sensory-neuron-cardiac systems remains limited. The following applications are therefore proposed experiments assembled from separate evidence streams: conditioned medium from hypoxic iPSC-cardiomyocytes could be applied to iPSC nociceptors, with ATP degradation, receptor blockade, or neutralizing antibodies used to test candidate mediators; conversely, sensory-neuron-conditioned medium could be assessed for effects on cardiomyocyte survival, contractility, or calcium handling. Any findings would require confirmation in direct co-culture and more mature multicellular systems.

The principal molecular pathways implicated in cardiac-related pain, together with their evidence in iPSC models and translational relevance, are summarized in Table 1.

## 3. iPSC Platform Technologies: Matching Complexity to Cardiac Pain Research Questions

Potential iPSC-based cardiac-pain research strategies span relatively scalable two-dimensional assays, neuro-cardiac co-cultures, three-dimensional cardiac tissues, and microfluidic devices. Available evidence differs substantially across these model classes; platform selection should match the biological question while accounting for throughput, cell provenance, and validation status.

### 3.1. Two-Dimensional Monocultures: High-Throughput Target Validation and Drug Screening

Two-dimensional iPSC-derived cardiomyocyte or nociceptor monocultures provide relatively high throughput and compatibility with established electrophysiology and imaging. Multiwell MEA assays have reported robust Z′-factors at or above 0.5 under selected seeding densities and inhibitor controls [35]. A separate TNF-α-sensitized calcium assay reported a modified Z′ of 0.56 using DMSO and lidocaine controls [63]. Calcium imaging with chemical or genetically encoded indicators supports population-level measurement of stimulus-evoked responses and concentration–response relationships [70,71]; application to a particular TRPV1 or P2X3 antagonist requires target-specific assay validation. Manual and automated patch-clamp electrophysiology can directly measure ion currents, action-potential parameters, and pharmacological modulation [72].

Two-dimensional platforms are particularly useful for cell-autonomous target validation. SCN9A knockout increases rheobase and reduces repetitive firing in human iPSC-derived nociceptors [29]. Future isogenic P2X3 knockout studies could test its contribution to ATP-evoked responses; current support comes from P2X3 reviews and rodent knockdown studies rather than direct human iPSC-knockout evidence [73,74]. iPSC-cardiomyocytes are also used in safety pharmacology: field-potential or action-potential duration and arrhythmic events can contribute to proarrhythmia assessment, although responses vary by cell line and ion-channel mechanism and are not a hERG-specific readout [75,76].

Two-dimensional monocultures cannot directly reproduce cross-cell-type paracrine or contact-dependent signaling. They also lack the three-dimensional tissue context and electrical-conditioning paradigms used to promote hPSC-cardiomyocyte maturation [77]. A compartmentalized platform combining hiPSC-cardiomyocytes with differentiated rat PC12 neurons demonstrated neuromodulation of cardiomyocyte calcium handling; the PC12 cells displayed mixed adrenergic and cholinergic markers and predominantly cholinergic secretion, limiting their interpretation as sympathetic neurons [78]. The same study also showed structural contacts between hiPSC-derived autonomic neurons and patient-matched hiPSC-cardiomyocytes. Accordingly, two-dimensional platforms are best used for initial target validation, ion-channel pharmacology, and primary screening, with multicellular questions transferred to appropriately validated co-culture or three-dimensional models.

### 3.2. Co-Culture Systems: Reconstructing Neuro-Cardiac Paracrine Signaling

Published human iPSC neuro-cardiac co-culture evidence includes validated sympathetic-neuron systems [20,22] and a compartmentalized study using hiPSC-derived neurons initially patterned toward cortical phenotypes that subsequently displayed mixed autonomic characteristics [21]; none used nociceptors. A cardiac nociceptor co-culture exposed to hypoxia, glucose deprivation, or acidosis is therefore a proposed configuration, not an established model. In such a system, MEA, patch clamp, and calcium imaging could quantify neuronal responses, while apyrase, neutralizing antibodies, or receptor antagonists could test candidate mediators. Separately, NGF-dependent modulation of P2X3 function has been demonstrated in mouse trigeminal sensory neurons [67]. References [17,18] characterize human iPSC-derived nociceptors [17,18] but do not establish a human cardiac nociceptor co-culture.

Transwell systems can separate two cell populations while allowing exchange of soluble factors [79]. Applying this format to human iPSC-derived sensory neurons and cardiomyocytes remains a proposed neuro-cardiac configuration. Separately, human iPSC-derived sensory neurons can release substance P after TRPV1 agonism [80], and sensory-neuron-derived CGRP modulates endothelial signaling in a non-cardiac co-culture [81]; effects on iPSC-cardiomyocytes remain to be tested. Human hPSC-derived sympathetic-neuron-cardiomyocyte systems demonstrate β-adrenergic modulation of beating [82], and patient-specific cardiac-neural microtissues model sympathetic contributions to CPVT-associated dysfunction [83]. Neither study establishes a human cardiac nociceptor model.

Compartmentalized microfluidic devices can physically separate neuronal cell bodies from target cells while permitting axonal extension, thereby preserving connectivity with fluidic isolation [23,84]. Microchannel electrodes can measure axonal conduction velocity and its change over time [85]. Such devices also support studies of retrograde axonal transport and signaling [86]. Their use for compartment-selective cardiac ischemic stress remains a prospective adaptation. Microfluidic neural systems require specialized fabrication and operational control; throughput and reproducibility depend on device design and consistency of axonal extension [87,88].

### 3.3. Three-Dimensional Organoids: Physiological Architecture and Innervation

Human cardiac organoids can be generated through guided self-organization of pluripotent-stem-cell aggregates or by assembling differentiated cardiac cell populations. Self-organizing hPSC cardiac organoids can develop chamber-like or regionally patterned features [24,26], whereas assembled hiPSC-derived cardiac microtissues can incorporate cardiomyocytes with supporting stromal populations and exhibit coordinated contractile activity [25]. Three-dimensional cardiac organoids and engineered microtissues can improve selected structural, electrophysiological, and metabolic maturation features relative to two-dimensional culture, but the degree of maturation is protocol-dependent [27]. Endothelial-network and perfusion strategies reported for cerebral organoids may improve nutrient transport [89] but require cardiac-specific validation. Sympathetic-neuron-innervated cardiac assembloids have been reported in a preprint, with TH-positive networks and increased beating after sympathetic stimulation [28]. Sensory–neuron incorporation remains prospective: scalable human PSC-derived nociceptors are available [90], cardiomyocyte-to-sympathetic-neuron NGF transfer has been shown in non-iPSC and tissue preparations [91], and ischemia-responsive cardiac afferents have been recorded in cats [92]. These separate findings do not establish a human sensory-innervated cardiac organoid.

Simulated hypoxia-reoxygenation can induce myocardial injury, oxidative stress, apoptosis, and impaired contractile function in human cardiac organoids [93]. In a separate primary murine DRG-cardiomyocyte system, sensory-neuron-conditioned medium improved cardiomyocyte survival during simulated ischemia–reperfusion, although the protective factor was not identified [94]. Extending that result to human iPSC co-innervated cardiac organoids remains prospective. Magnetic torque stimulation has been reported to increase maturation-related and vascular-associated gene expression and improve structural and functional features in hESC-derived cardiovascular organoids [95].

Reviews identify low throughput, batch variability, labor-intensive protocols, and incomplete maturation as limitations of cardiac organoid platforms [96,97]. Reproducible dense sensory innervation and functional connectivity have not been established in the cardiac organoids discussed here. Given these constraints, organoids are best viewed as platforms for hypothesis-driven cardiac-development and ischemia-injury studies [93,96,97,98]. Their use for patient-specific refractory-angina modeling or late-stage cardiac-pain analgesic validation remains prospective and depends on reproducible sensory innervation.

### 3.4. Microfluidic Organ-on-Chip: Precision Control and Multi-Organ Integration

Microfluidic heart-on-chip platforms combine human cardiac cells with defined flow, mechanical stimulation, or integrated functional readouts. Reported examples include integrated hiPSC-cardiac tissues, multicellular heart-on-chip systems, and functional three-dimensional cardiac microtissues [99,100,101]. The presence of endothelialized channels, cyclic loading, perfusion, and long-term culture varies by device design and should not be assumed for every platform.

Hypoxia-reoxygenation or acute-hypoxia protocols have been implemented in cardiac microphysiological systems to model selected aspects of ischemic injury [102,103]. Integration of sensory neurons is a prospective strategy for measuring neuro-cardiac paracrine signaling under controlled ischemic stress; fully validated human sensory-neurocardiac ischemia–reperfusion chips have not yet been established.

Heart-on-chip studies have modeled myocardial responses to hypoxia [102], monitored electrophysiology under acute hypoxia [103], and evaluated endothelial extracellular-vesicle cardioprotection during ischemia–reperfusion injury [104]. Multi-organ chips can connect cardiac and metabolic tissue compartments for systemic toxicity studies [105], whereas fluidically coupled vascularized organ chips have been used to model pharmacokinetic responses [106]. Their relevance to cardiac-pain pharmacology, particularly after incorporation of sensory neurons, remains prospective. Heart-on-chip systems typically require specialized fabrication, fluidic control, and analytical expertise and generally offer lower throughput than plate-based assays [107]. They are therefore most appropriate for mechanistic studies and later-stage validation when physiological control justifies operational complexity.

### 3.5. Strategic Platform Selection for Cardiac Pain Research

Platform choice should match the biological question to the minimum complexity needed for a meaningful answer. Testing whether SCN9A/Nav1.7 perturbation alters nociceptor excitability is well-suited to two-dimensional gene-edited iPSC-nociceptor cultures analyzed by electrophysiology [29]. Phenotypic screening can use multiwell MEA assays [35,63] or calcium imaging [70,71]; target-specific P2X3 or TRPV1 programs require validated agonist and antagonist controls rather than assuming that a general screening platform establishes target-specific performance.

Mechanistic questions concerning cardiomyocyte-derived mediators can be addressed in proposed conditioned-medium or direct co-culture experiments, using mediator measurements together with apyrase, neutralizing antibodies, or selective receptor antagonists. A patient-specific cardiac-neural microtissue study has demonstrated sympathetic contributions to an inherited arrhythmia phenotype [83]; it did not use nociceptors. Applying related multicellular designs to cardiac-pain mechanisms remains prospective.

Compartmentalized microfluidic chambers permit direct tracking of neurotrophic-factor transport along primary neuronal axons [108]. Their use for axon-selective cardiac-ischemia experiments remains prospective. Organ-on-chip systems are best considered for advanced mechanistic studies and later-stage lead validation, where controlled perfusion, mechanical loading, and multicellular complexity justify lower throughput [107]. Strategic progression from scalable two-dimensional assays to co-culture, three-dimensional, or microfluidic systems should be driven by the biological question. 

The key characteristics, strengths, and limitations of 2D monoculture, co-culture, 3D organoid, and organ-on-chip platforms are compared in Table 2.

## 4. Translating iPSC Insights to Cardiac Pain Therapeutics

**Figure 2:**
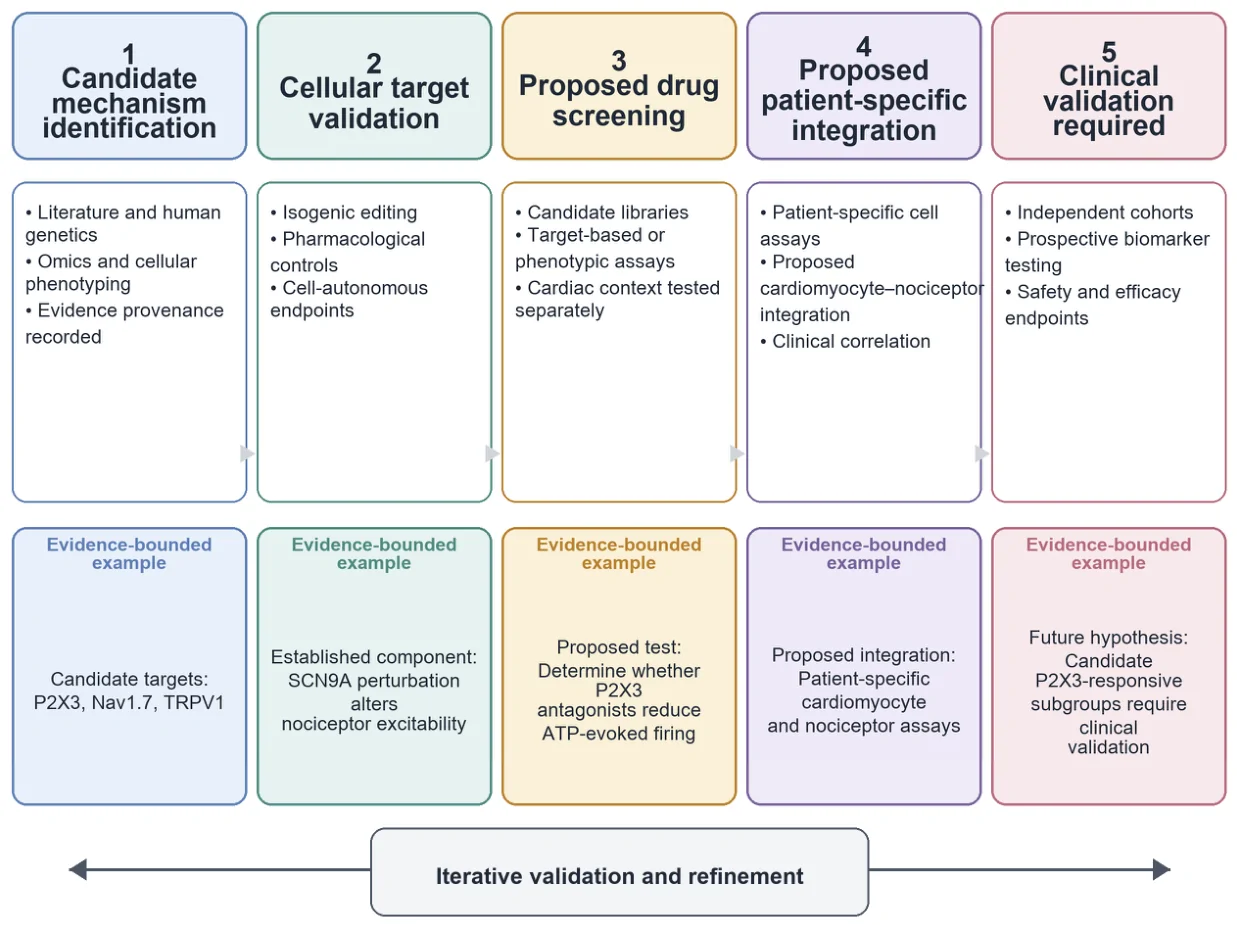
Proposed translational framework for future iPSC-based cardiac-pain research.

This section considers potential uses of iPSC-derived cells in cardiac-pain therapeutic research, including target validation, patient-specific disease modeling, and pharmacological screening (Figure 2). Because direct human sensory-neuron-cardiac validation remains limited, each application is evaluated according to whether it is established in the cited model or remains a proposed translation from separate evidence streams.

Conceptual workflow for future iPSC-based cardiac-pain research. Patient-derived somatic cells may be reprogrammed and differentiated into cardiomyocytes, nociceptors, and, where relevant, sympathetic or endothelial lineages. Available cell types can be studied in assays matched to the question, while sensory-neuron-innervated cardiac organoids and sensory-neurocardiac ischemia-on-chip configurations require direct validation. Hypoxia, low-glucose exposure, or inflammatory stimulation may be used as defined experimental stressors. Functional and molecular measurements could generate candidate targets, pharmacological-response phenotypes, or subgroup hypotheses for subsequent preclinical and clinical testing; the workflow does not imply that an iPSC-based cardiac-pain treatment-selection pipeline is already validated.

### 4.1. Target Validation in Human Cellular Contexts: P2X3 and Nav1.7 Case Studies

P2X3 and Nav1.7 are well-studied nociception-relevant sensory targets that illustrate distinct translational challenges. Human iPSC-derived sensory–neuron studies demonstrate ATP- or α,β-meATP-evoked calcium responses [30,31], while MEA and high-content studies establish general functional and screening capabilities [18,35,36,63]. P2X3-containing receptors are supported as pain-relevant targets by the broader pharmacological literature [110], but direct P2X3-antagonist validation in a human cardiac-pain model remains absent.

Gefapixant (MK-7264; AF-219), an antagonist of P2X3-containing receptors, has provided clinical proof of concept for P2X3/P2X2/3 blockade in refractory or unexplained chronic cough. In the Phase III COUGH-1 and COUGH-2 trials, 45 mg twice daily significantly reduced 24 h cough frequency relative to placebo, whereas 15 mg twice daily did not meet the primary efficacy endpoint [37]. Taste-related adverse events were common [37]. Gefapixant is authorized in the European Union and Japan for chronic-cough indications [111,112]. Human iPSC-derived sensory neurons show ATP- or α,β-meATP-evoked calcium responses [30,31], while MEA and high-content platforms support general functional screening [18,35,36,63]. Direct evidence that gefapixant suppresses responses induced by ischemic human iPSC-cardiomyocyte-conditioned medium has not been reported; such a co-culture experiment remains a proposed cardiac-pain application.

Human genetic studies establish SCN9A/Nav1.7 as a determinant of pain phenotypes. Biallelic loss-of-function variants can cause congenital inability to experience pain [51], whereas gain-of-function variants are associated with inherited pain disorders including erythromelalgia and paroxysmal extreme pain disorder [52]. These observations establish biological relevance but do not by themselves establish that pharmacological Nav1.7 inhibition will be clinically analgesic. In a randomized trial in painful diabetic peripheral neuropathy, PF-05089771 did not produce a statistically significant reduction in pain relative to placebo [65]; broader translational limitations have been reviewed [53].

Human iPSC-derived nociceptors may help investigate Nav1.7-dependent excitability, drug responses, and compensatory ion-channel mechanisms in a human genetic background. Differentiation studies establish nociceptor identity and functionally relevant sodium-channel repertoires [54,113], whereas an isogenic SCN9A study demonstrates gene-edited changes in excitability [29]. Whether species pharmacology, neuronal maturation, central mechanisms, or compensation by other channels accounts for clinical failures remains unresolved and requires direct study in standardized human models [52,53,114].

CRISPR-based editing in iPSC-derived nociceptors offers a practical framework to test these mechanisms systematically. For example, isogenic SCN9A loss-of-function lines, alone or combined with perturbation of SCN10A, SCN11A, or TRPV1, could determine whether multi-target strategies suppress nociceptor excitability more effectively than selective Nav1.7 inhibition. Such experiments should be interpreted as prospective mechanistic studies rather than as established evidence that combined Nav1.7/Nav1.8 or Nav1.7/TRPV1 inhibition will provide superior clinical analgesia.

Together, P2X3 and Nav1.7 illustrate the value and limits of human iPSC nociceptor platforms. Validated component technologies include isogenic SCN9A manipulation [29] and MEA or high-content screening [35,36,63]. Their integration with patient-specific, ischemia-stressed cardiomyocyte co-cultures remains prospective; it should not be described as an established route to cardiac-pain therapy.

### 4.2. Patient-Specific Disease Modeling: Refractory Angina and Microvascular Angina

Refractory angina is generally defined as chronic chest pain caused by myocardial ischemia in patients receiving maximal medical therapy who are not suitable for further coronary revascularization. Despite improvements in medical treatment and risk-factor management, refractory angina remains associated with substantial impairment in quality of life, high healthcare use, and limited therapeutic options [115,116].

The mechanisms underlying persistent angina are heterogeneous. Coronary microvascular dysfunction contributes to myocardial ischemia in some patients [117,118], while a contribution of interindividual sensory processing to symptom severity remains plausible but incompletely established. Patient-specific iPSC models may provide a way to examine selected cellular mechanisms. Somatic cells can be reprogrammed to iPSCs and differentiated into patient-specific cardiomyocytes [119,120]; sensory–neuron differentiation uses separate, specialized protocols [113,121]. These cell types retain the donor genetic background, but they do not reproduce the patient’s complete clinical or environmental context.

In principle, patient-derived iPSC nociceptors could be evaluated for basal electrophysiological properties, responses to ATP, inflammatory mediators, or conditioned medium from hypoxia-stressed cardiomyocytes, and expression of pain-associated genes such as SCN9A, TRPV1, NTRK1, and purinergic receptor genes. Parallel evaluation of patient-derived cardiomyocytes could assess susceptibility to hypoxic or metabolic stress. Such experiments may help determine whether altered cardiomyocyte stress responses, sensory–neuron excitability, or their interaction contributes to persistent angina symptoms. Direct evidence linking these in vitro phenotypes to refractory-angina severity or treatment response is currently limited.

Microvascular angina is associated with coronary microvascular dysfunction and is an important contributor to ischemia with no obstructive coronary artery disease (INOCA). It is characterized by anginal symptoms and objective evidence of myocardial ischemia attributable to abnormal coronary microvascular function, commonly reflected by impaired vasodilatory capacity, reduced coronary flow reserve, and/or microvascular spasm [117,118,122].

iPSC-derived cardiomyocytes and endothelial cells may be incorporated into two-dimensional co-cultures, three-dimensional cardiac tissues, or microfluidic systems to investigate cellular consequences of microvascular dysfunction. These platforms could be used to examine endothelial nitric-oxide signaling, endothelin-related responses, cardiomyocyte metabolic stress, and ischemia-associated mediator release under controlled conditions. Incorporation of iPSC-derived nociceptors would further allow testing of whether endothelial or cardiomyocyte stress signals alter sensory–neuron excitability. However, such multicellular models should currently be regarded as hypothesis-testing systems rather than established models that reproduce the full clinical phenotype of microvascular angina [118,122,123].

A longer-term objective is to integrate iPSC-derived functional measurements with clinical, genomic, and transcriptomic data. For example, patient-derived nociceptors could be profiled for responses to ATP, inflammatory mediators, or cardiomyocyte-conditioned medium, and these results could be compared with pain characteristics, clinical imaging data, and molecular signatures. This approach may identify reproducible cellular phenotypes associated with distinct symptom patterns, but no validated iPSC-based molecular classification or treatment-selection framework currently exists for refractory angina or microvascular angina.

Patient-specific iPSC platforms may also be explored for silent myocardial ischemia, in which ischemia or infarction occurs with absent or reduced symptoms [3]. Comparative iPSC-sensory-neuron studies of individuals with silent versus symptomatic ischemia are prospective and have not yet established causal mechanisms. Proposed involvement of SCN9A, P2RX3, or TRPV1 therefore remains hypothetical.

### 4.3. Challenges and Path Forward

Despite substantial progress, several challenges continue to limit the translational application of iPSC-based platforms. Cellular maturation remains a primary concern. Compared with adult ventricular cardiomyocytes, iPSC-derived cardiomyocytes commonly retain fetal-like structural, electrophysiological, and metabolic characteristics, including relatively immature sarcomere organization, less negative resting membrane potentials, and incomplete metabolic maturation [27,124,125]. Similarly, iPSC-derived sensory neurons acquire nociceptor-like properties progressively during differentiation and culture, but they may not fully reproduce the electrophysiological, molecular, and functional characteristics of adult primary human dorsal root ganglion neurons [64,126]. These maturation differences should be considered when interpreting pharmacological endpoints, including concentration–response relationships, maximal drug effects, and cross-platform comparisons [127].

Maturation strategies remain an active area of development. For iPSC-derived cardiomyocytes, prolonged culture, electrical pacing, mechanical loading, three-dimensional tissue assembly, and metabolic conditioning can improve structural and functional maturation, although adult-like properties are not fully reproduced [27,124,125]. In sensory-neuron systems, prolonged culture and optimization of differentiation conditions can enhance nociceptor functionality, but the optimal maturation approach depends on the cell line, differentiation protocol, and intended experimental endpoint [17,54,64]. Rather than precluding their utility, the immature state of iPSC-derived cells should be transparently acknowledged and incorporated into experimental design, data interpretation, and assay validation [128].

Batch-to-batch and donor-to-donor variability pose additional challenges for reproducibility. Sources of variation include donor genetic background, reprogramming history, differentiation efficiency, cell-composition heterogeneity, culture conditions, and assay-analysis methods [121,128,129]. Functional readouts, including responses to capsaicin, ATP, inflammatory mediators, or depolarizing stimuli, may therefore vary across cell lines and differentiation batches. Mitigation strategies include predefined quality-control criteria, characterization of cellular identity and purity, use of standardized reference iPSC lines where appropriate, replication across independent donor lines, and inclusion of batch and donor effects in statistical analyses [130,131]. Linear mixed-effects models and related approaches can help distinguish treatment-associated effects from variability attributable to donor identity or differentiation batch.

Standardization is required not only for cell production but also for assay design and data analysis. Differences in stimulus concentration, exposure duration, media composition, culture age, recording conditions, and analytical thresholds may substantially influence functional outcomes [36,54,64,113]. Existing initiatives in cardiac safety pharmacology, including the Comprehensive in vitro Proarrhythmia Assay (CiPA), provide a useful regulatory-science precedent for assay standardization and multicentre validation [132,133,134]. However, equivalent consensus protocols, reference compounds, and performance criteria remain to be established for iPSC-derived nociceptor assays. Precompetitive collaborations among academic groups, biotechnology companies, pharmaceutical companies, and regulatory scientists could facilitate the development of common protocols and reproducible benchmark datasets.

Regulatory use of iPSC-derived data is evolving. The FDA Modernization Act 2.0 changed U.S. statutory language so that nonclinical evidence need not be limited to animal tests; the cited commentaries discuss human cell-based and computational approaches as possible components [135,136]. This change does not constitute regulatory acceptance of any specific iPSC assay. Regulatory value depends on a defined context of use and evidence of biological relevance, technical robustness, reproducibility, and fitness for purpose [137]. CiPA provides a cardiac-safety standardization precedent, but it does not validate a nociceptor assay [76,132].

Trials of gefapixant in refractory or unexplained chronic cough demonstrate clinical activity of P2X3/P2X2/3 blockade, although average benefits were modest and taste-related adverse events were common [37,61,62]. These findings do not establish efficacy in cardiac pain or validate an iPSC-derived nociceptor assay for cardiac-pain drug development. Human iPSC-derived nociceptor platforms may nevertheless support studies of ATP-dependent signaling, receptor pharmacology, and interindividual sensory–neuron responses before progression to more complex preclinical and clinical studies.

Near-term opportunities center on integrating iPSC functional assays with complementary technologies. Multi-omics may identify molecular correlates of neuronal or cardiomyocyte phenotypes, but reference [138] supports this strategy by analogy from neurodegeneration rather than by direct cardiac-pain evidence. High-content neuronal profiling and image-based machine-learning studies show that multidimensional features can be extracted from cellular assays [139,140]. Their ability to predict cardiac-pain drug efficacy or treatment selection requires independent external validation.

Multi-organ chips can connect cardiac and metabolic tissue compartments for selected systemic-toxicity studies [105], whereas fluidically coupled vascularized organ chips have been used to model pharmacokinetic responses [106]. Their relevance to cardiac-pain pharmacology, particularly after incorporation of sensory neurons or dorsal-root-ganglion-like components, remains prospective. Such integrated systems require validation of tissue maturation, inter-tissue communication, flow conditions, and assay reproducibility before application to cardiac-pain drug development.

By analogy with stratified approaches in neuropathic pain [141] and general iPSC pain-modeling frameworks [142], patient-derived iPSC phenotypes could be explored for candidate subgroup discovery. Associations among cellular responses, symptoms, imaging, and genetic variation could generate hypotheses, but no cited study validates an iPSC biomarker or treatment-selection framework for cardiac pain. Prediction of individual analgesic responses or clinical-trial success remains unestablished.

Human cellular pain models span directly reprogrammed nociceptors [143] and increasingly scalable iPSC-derived sensory-neuron assays [18,35,36,63]; broader iPSC and human-DRG opportunities and limitations have been reviewed [142]. Translation to cardiac pain will require standardized assays, multicenter validation, and explicit linkage between in vitro endpoints and clinical outcomes before these systems can support regulatory decisions, patient stratification, or treatment selection. 

Candidate analgesic targets, their corresponding mechanisms, development stages, iPSC model evidence, and current clinical status are summarized in Table 3.

## 5. Conclusions and Future Perspectives

Cardiac-related pain remains an important unmet clinical need. Refractory angina is associated with persistent symptoms and impaired quality of life despite guideline-directed medical therapy and, in many patients, limited options for further revascularization [115,116]. Silent myocardial ischemia and unrecognized myocardial infarction may delay clinical recognition and are associated with adverse clinical outcomes [3]. Species differences in pain biology and ion-channel phenotype can limit translation from animal models [114,152]. Human iPSC systems therefore provide a complementary platform for studying defined peripheral cellular mechanisms [64,113,121,142].

This review has summarized three evidence streams relevant to future iPSC-based cardiac-pain research. Human iPSC-derived sensory neurons demonstrate responses to nociceptive agonists and inflammatory stimuli in non-cardiac experimental contexts [30,31,63,64,113]. Primary adult human sensory neurons provide complementary evidence for inflammatory sensitization [126]. Human iPSC-derived cardiomyocytes and cardiac tissues model selected responses to hypoxia or ischemia-like stress [93,98,102,103]. These separate capabilities support, but do not yet constitute, a validated human cardiac-pain model. Established readouts include electrophysiology and MEA [18,29,35], calcium imaging [30,31], gene-expression or transcriptomic profiling [17], high-content physiological profiling [139], neuropeptide release in human iPSC-derived sensory neurons [80], and conditioned-medium effects in a primary murine DRG-cardiomyocyte system [94].

Section 3 evaluated platforms from two-dimensional monocultures to co-cultures, three-dimensional tissues, and microfluidic devices. Multiwell iPSC-nociceptor assays have published screening and performance data [35,63]. Published human neuro-cardiac co-cultures include validated sympathetic-neuron systems [20,22] and a study using neurons with mixed autonomic characteristics [21]; none used nociceptors. Mediator-specific causal evidence in human sensory–neuron–cardiac co-cultures remains limited. Three-dimensional cardiac tissues can incorporate multiple cardiac lineages [27,98], while organ-on-chip reviews describe broader engineered-tissue strategies [153]; mature sensory innervation is not routinely reproduced. Microfluidic platforms provide spatial control, axonal compartmentalization, or perfusion depending on device design [23,84,99,100,123]. Platform selection should therefore follow the specific biological question rather than an assumed linear hierarchy of model fidelity.

Section 4 considered target validation, pharmacological screening, and patient-derived disease modeling. Trials of gefapixant in refractory or unexplained chronic cough demonstrate clinical activity of P2X3/P2X2/3 blockade, but do not establish efficacy in cardiac pain [37,61,62]. Selective Nav1.7 inhibition with PF-05089771 did not show consistent analgesic efficacy in painful diabetic neuropathy or experimental-pain studies [53,65,66]. iPSC-derived peripheral-neuron assays can investigate cell-autonomous mechanisms but do not reproduce spinal or supraspinal pain processing. Patient-specific iPSC models may help investigate interindividual cellular variation, but their ability to predict symptom severity or treatment response remains unestablished [121,128,141,142].

Several limitations require continued attention. iPSC-derived cardiomyocytes commonly retain immature structural, electrophysiological, and metabolic features [27,125], while iPSC-derived sensory neurons can retain developmental differences in channel repertoire and function relative to adult human DRG [17,54,64,121]. These limitations affect quantitative pharmacological endpoints and cross-model comparisons. Batch and donor variability also arise from genetic background, reprogramming history, differentiation efficiency, culture conditions, cellular composition, and assay analysis [128,129,130,131]. Robust studies should use predefined quality-control criteria, independent donor lines or appropriate reference lines, and statistical models that account for donor and batch effects [128,130,131].

iPSC-based systems also have inherent biological boundaries. They do not reproduce the integrated spinal and supraspinal circuitry involved in visceral-somatic convergence, referred pain, autonomic regulation, and central pain modulation. Accordingly, their principal value lies in investigating peripheral cellular mechanisms, identifying candidate therapeutic targets, and generating human-relevant pharmacological data that complement rather than replace animal studies, ex vivo human tissues, and clinical research [4,142,154,155]. 

Near-term opportunities include integration of iPSC functional assays with multi-omics and high-content phenotyping. Reference [138] supports multi-omics by methodological analogy from neurodegeneration, while references [139,140] support high-content phenotyping; direct cardiac-pain validation remains limited [138,139,140]. Multi-organ chips can connect cardiac and metabolic tissue compartments for systemic-toxicity studies [105], whereas fluidically coupled vascularized organ chips can model pharmacokinetic responses [106]. Their relevance after incorporation of sensory neurons remains prospective.

By analogy with stratified approaches in neuropathic pain [141] and general iPSC pain-modeling frameworks [142], patient-derived iPSC phenotypes could be explored for candidate subgroup discovery. No cited study validates an iPSC biomarker or treatment-selection framework for cardiac pain; any proposed subgroup requires independent replication and prospective clinical validation.

Regulatory pathways for iPSC-derived evidence are evolving. The FDA Modernization Act 2.0 changed U.S. statutory language so that nonclinical evidence need not be limited to animal tests; the cited commentaries discuss human cell-based and computational approaches as possible components [135,136]. This change does not constitute regulatory acceptance of any specific iPSC assay. Assay use requires a defined context, biological relevance, technical robustness, reproducibility, and fit-for-purpose performance [137]. CiPA provides a cardiac-safety standardization precedent rather than validation of a nociceptor assay [76,132]. The European Medicines Agency provides a qualification pathway for novel methodologies within a defined context of use [156].

Cardiac pain remains a challenging clinical problem, and iPSC-based platforms offer a human-relevant approach for studying peripheral mechanisms that are difficult to interrogate in conventional animal models. Continued progress will depend on improved cellular maturation, reproducible differentiation and assay protocols, multicenter validation, transparent reporting of model limitations, and prospective linkage of in vitro findings to clinical outcomes [27,76,128,130,132]. Through sustained collaboration among stem-cell biologists, cardiologists, pain researchers, bioengineers, medicinal chemists, data scientists, clinical trialists, regulatory scientists, and patient advocates, iPSC-based systems may contribute to more mechanism-informed and patient-informed approaches to cardiac-pain research and therapeutic development.

## Figures and Tables

**Table 1:** Key molecular pathways implicated in cardiac-related pain and their modeling in iPSC systems. The table summarizes candidate algogenic mechanisms, their principal mediators, proposed roles in cardiac pain, corresponding model evidence, and translational relevance. The “iPSC Model Evidence” column indicates whether the supporting evidence derives from human iPSC-derived sensory neurons, primary human cells, animal models, or proposed cardiac co-culture experiments. These distinctions prevent pathway plausibility from being interpreted as direct validation in a human iPSC-derived cardiac-pain model.

Pathway	Key Molecules (Examples)	Role in Cardiac Pain	iPSC Model Evidence	Translational Relevance
Purinergic signaling	ATP, P2X3, P2X2/3	ATP released during ischemia can activate cardiac afferent pathways through purinergic receptors [5,6]	Human iPSC-derived sensory neurons show ATP- or α,β-meATP-evoked calcium responses [30,31], while MEA studies establish quantifiable pharmacologically evoked firing [18,35,36]. Suppression of responses to cardiac-ischemia-conditioned medium by P2X3 antagonists has not been demonstrated.	Gefapixant has clinical activity in refractory or unexplained chronic cough [37,61,62]; its value in cardiac pain remains untested
Inflammatory signaling	Bradykinin, endothelin-1, TNF-α, IL-1β, NGF	Vasoactive and inflammatory mediators can alter sensory-neuron signaling in model-dependent contexts [39,40,41,42]	Rodent sensory-neuron studies report IL-1β-related excitability [40], NGF-associated TRPV1 upregulation [41], and TNF-α enhancement of P2X3 currents [42]. Human iPSC nociceptor assays can test inflammatory phenotypes [63,64], but direct cardiac co-culture evidence remains limited.	Candidate pathways for mechanistic testing; no established anti-inflammatory analgesic strategy for cardiac pain
Ion channel signaling	Nav1.7, Nav1.8, TRPV1, TRPA1, ASICs	Voltage-gated sodium, TRP, and acid-sensing channels regulate nociceptor excitability or stimulus transduction [8,9,48,55]	SCN9A perturbation alters excitability in human iPSC-derived nociceptors [29]; sodium-channel and TRPV1 function have been characterized in human iPSC nociceptors [54,55].	Human genetics support Nav1.7 in pain biology [50,51,52], but PF-05089771 showed inconsistent analgesic efficacy [53,65,66]; TRP- and ASIC-directed cardiac-pain approaches remain untested
Oxidative stress	ROS, NOX2, Glutathione	A proposed mechanism is sensitization of nociceptors through redox-sensitive channels; direct relevance to cardiac pain remains unproven	Hypoxia-related TRPA1 sensitization by reactive oxygen species was demonstrated in a mouse hindlimb ischemia–reperfusion model [44]; direct human cardiac iPSC-nociceptor evidence is absent.	Antioxidant and redox-modulating approaches remain exploratory for cardiac-pain treatment
Neurotrophic signaling	NGF, BDNF, TrkA, TrkB	Neurotrophins can sensitize nociceptors, and cardiac injury can alter BDNF or NGF signaling in species- and model-specific contexts [47,56,57,58,59]	NGF-dependent nociceptor sensitization and P2X3 modulation are established in non-cardiac or mouse sensory-neuron contexts [47,67]; direct human cardiac iPSC-nociceptor evidence is absent.	Anti-NGF therapy has been evaluated in non-cardiac osteoarthritis pain [68,69]; relevance to cardiac pain requires direct validation

**Table 2:** Comparison of experimental platforms relevant to future iPSC-based cardiac-pain research. Two-dimensional monocultures, co-culture systems, three-dimensional organoids (including innervated assembloids), and microfluidic organ-on-chip platforms are compared in terms of cellular composition, structural and microenvironmental features, scalability, and principal experimental applications. The characteristics shown represent typical implementations reported in the literature and may vary according to the specific protocol and device design. This comparison is intended to guide platform selection by balancing mechanistic resolution, physiological fidelity, and translational relevance.

Feature	2D Monoculture 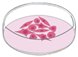	Co-Culture 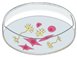	3D Organoid 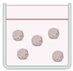	Organ-on-Chip 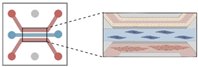
**Cell Type Diversity**	Typically, one predominant iPSC-derived cell type	Two or more defined cell populations; cellular provenance depends on study design	Multiple cardiac and supporting cell populations generated by guided differentiation or cellular assembly	Multiple cell types, potentially including endothelial and immune cells, depending on chip design
**Structure**	Planar monolayer	Planar co-culture, or simple scaffold-supported configuration	Self-organized or assembled three-dimensional tissue	Three-dimensional tissue within fluidic channels, with defined compartmentalization
**Cell–Cell Interactions**	Predominantly homotypic, planar contact	Direct and indirect interactions	Multidirectional 3D cell–cell interactions within the modeled tissue; network completeness depends on composition and maturation	3D interactions and fluid-mediated paracrine signaling
**Cell–Matrix Interactions**	ECM-coated surface (e.g., Matrigel, laminin, fibronectin)	ECM-coated surface or simple hydrogel/scaffold system	Cells embedded in or assembled with hydrogels or engineered matrices	Matrix composition can be defined within tissue chambers or microchannels
**Fluid Shear Stress**	Absent in static culture	Absent in static culture	Generally absent, unless integrated with a perfusion system	Controlled perfusion and fluid shear can be introduced in perfused designs
**Vascularization Capacity**	Typically absent unless endothelial cells are deliberately added	Endothelial co-culture is possible but generally lacks functional perfusion	Endothelial cells or vascular-like networks can be incorporated, but perfused vasculature is not yet routinely achieved	Perfusable endothelialized channels can be incorporated in selected chip designs
**Immune Component Integration**	Possible via deliberate immune-cell addition, but not routinely incorporated	Immune cells can be added to interrogate defined paracrine or contact-dependent effects	Immune-cell incorporation is feasible but increases cellular and batch complexity	Controlled introduction or perfusion of immune cells is possible in selected chip designs
**Long-Term Culture Capacity**	Days to weeks; protocol-dependent	Days to weeks; protocol-dependent	Usually weeks; longer culture is protocol-dependent	Days to weeks; device-dependent
**Throughput**	Relatively high	Moderate	Relatively low	Low to moderate; design-dependent
**Real-Time Monitoring**	Generally compatible with calcium imaging, MEA, and patch-clamp	Compatible with imaging and electrophysiology, though cell-type-specific readouts may require labeling	May require specialized imaging, optical reporters, or embedded sensors due to tissue thickness and optical limitations	High potential for continuous monitoring, though implementation is technically demanding
**Technical Complexity/Cost**	Low	Low to moderate	Moderate to high	High
**Principal Strengths**	Scalability, reproducibility, genetic tractability, and compatibility with high-content screening	Permits controlled study of paracrine or contact-dependent interactions; mediator-specific testing in human sensory-cardiac systems remains prospective	Greater tissue architecture, multicellular context, and improved maturation relative to 2D culture	Potential for spatial or temporal control, perfusion, mechanical stimulation, and compartment-specific perturbation, depending on device design
**Major limitations**	Lacks paracrine crosstalk, multicellular architecture, and physiological maturation cues	Incomplete tissue-level organization; cell-ratio and interface variability	Batch-to-batch variability, incomplete maturation, diffusion-limited cores, and low throughput	Device fabrication complexity, low scalability, and need for specialized fluidic/analytical expertise
**Applications**	Target validation and screening [18,29,35,63,72]	Autonomic neuro-cardiac models [20,21,22,82,83]; sensory-cardiac blockade studies remain prospective	Cardiac organoid studies of development and ischemia-like injury [24,26,93,98]; sensory-innervated cardiac-pain organoids remain prospective	Axonal compartmentalization studies [23,84,85,86,87,88] and separate cardiac perfusion or hypoxia studies [99,100,101,102,103,104,107,109]; integrated sensory-cardiac ischemia chips remain prospective

**Table 3:** Candidate analgesic targets relevant to future cardiac-pain research: model evidence and clinical-development status. The table presents examples of candidate analgesic targets relevant to nociceptor biology, including human iPSC-derived sensory-neuron evidence where available and clinical evidence from non-cardiac pain or sensory indications. It distinguishes target biology, compound-specific development evidence, direct human iPSC findings, and untested cardiac-pain applications. The absence of direct evidence is explicitly indicated for anti-NGF therapies, resiniferatoxin (RTX), ASIC-directed approaches, and sensory–cardiac configurations.

Target	Mechanism	Representative Drugs/Strategies	Development Stage	iPSC Model Findings	Clinical Outcome/Status
P2X3 receptor	ATP-gated P2X3 receptor expressed by a subset of sensory neurons [144].	Gefapixant (MK-7264)	Authorized in the EU and Japan (chronic cough)	Human iPSC-derived sensory neurons show ATP- or α,β-meATP-evoked calcium responses [30,31], and ATP-evoked firing has been demonstrated by patch clamp and MEA [36]. Gefapixant antagonism at P2X3 and P2X2/3 receptors has been characterized in recombinant systems [145]. Direct cardiac iPSC co-culture evidence has not been reported.	Authorized in the EU for refractory or unexplained chronic cough and in Japan for refractory chronic cough [37,61,62,111,112]; cardiac-pain efficacy is untested.
Na_v_1.7 channel	Voltage-gated sodium channel; sets nociceptor firing threshold [52].	PF-05089771	Tested in randomized clinical and experimental-pain studies	CRISPR-edited iPSC nociceptors support a role for SCN9A in excitability [29]. Biallelic loss-of-function variants can cause congenital inability to experience pain [51], whereas gain-of-function variants are associated with inherited pain disorders [52].	The cited studies did not demonstrate consistent analgesic efficacy with PF-05089771 [65,66]; cardiac-pain efficacy is untested.
NGF–TrkA axis	NGF signaling through TrkA contributes to nociceptor sensitization [47]; fasinumab is an anti-NGF antibody rather than a TrkA antagonist.	Fasinumab (anti-NGF antibody)	Evaluated in a phase IIb/III trial for osteoarthritis pain [68]	Direct functional validation of anti-NGF therapy in human iPSC-derived nociceptors or cardiac-nociceptor co-cultures was not identified in the evidence reviewed here.	Fasinumab showed analgesic efficacy signals in osteoarthritis, with important joint-safety concerns [68]. Broader anti-NGF development and safety considerations were reviewed previously [69]; cardiac-pain efficacy is untested.
TRPV1 channel	Capsaicin- and heat-activated ion channel involved in nociceptive signaling [146].	Capsazepine (preclinical pharmacological tool); other TRPV1 antagonists (class evidence)	Capsazepine: preclinical; antagonist-class development limited by mechanism-related adverse effects [147]	Heat- and capsaicin-evoked TRPV1 responses have been demonstrated in human iPSC-derived sensory neurons [148].	TRPV1-antagonist development has faced hyperthermia and impaired noxious-heat sensation [147]; cardiac-pain efficacy is untested.
TRPV1+ cells	RTX can desensitize or ablate these cells [147].	Resiniferatoxin (RTX)	A Phase I study was registered for severe refractory cancer pain [149]	Direct cardiac iPSC-nociceptor evidence has not been established.	A Phase I study of intrathecal RTX for severe refractory cancer pain was registered (NCT00804154) [149]; the registry citation does not establish efficacy, and cardiac-pain efficacy is untested.
ASICs	Acid-sensing ion channels; mediate responses to tissue acidosis [150].	APETx2 and amiloride (preclinical ASIC-directed approaches) [151]	Preclinical	References [150,151] establish ASIC pain biology but do not provide direct functional validation in human iPSC-derived nociceptors.	References [150,151] support ASIC/ASIC3 pain biology and experimental inhibition, but do not establish clinical analgesic efficacy; cardiac-pain efficacy is untested.

## Data Availability

No new data were created or analyzed in this study. Data sharing is not applicable to this article.

## Abbreviation

iPSC, induced pluripotent stem cell; ATP, adenosine triphosphate; ROS, reactive oxygen species; NGF, nerve growth factor; IL-1β, interleukin-1β; P2X3, P2X purinoceptor 3; TRPV1, transient receptor potential vanilloid 1; Nav1.7, voltage-gated sodium channel 1.7; MEA, multielectrode array; CNS, central nervous system.
